# Supplementary material for: Synthetic mammalian pattern formation driven by differential diffusivity of Nodal and Lefty
Source: Nat Commun. 2018 Dec 21;9:5456. doi: 10.1038/s41467-018-07847-x (PMC6303393; doi:10.1038/s41467-018-07847-x)
Supplement: Supplementary file 1 — Supplementary Information [file 41467_2018_7847_MOESM1_ESM.pdf]

Supplementary Information

Synthetic mammalian pattern formation driven by differential Diffusivity of Nodal and Lefty

Sekine et al.

This file includes:

Supplementary Figures 1 to 10

Supplementary Tables 1

Supplementary Figure 1

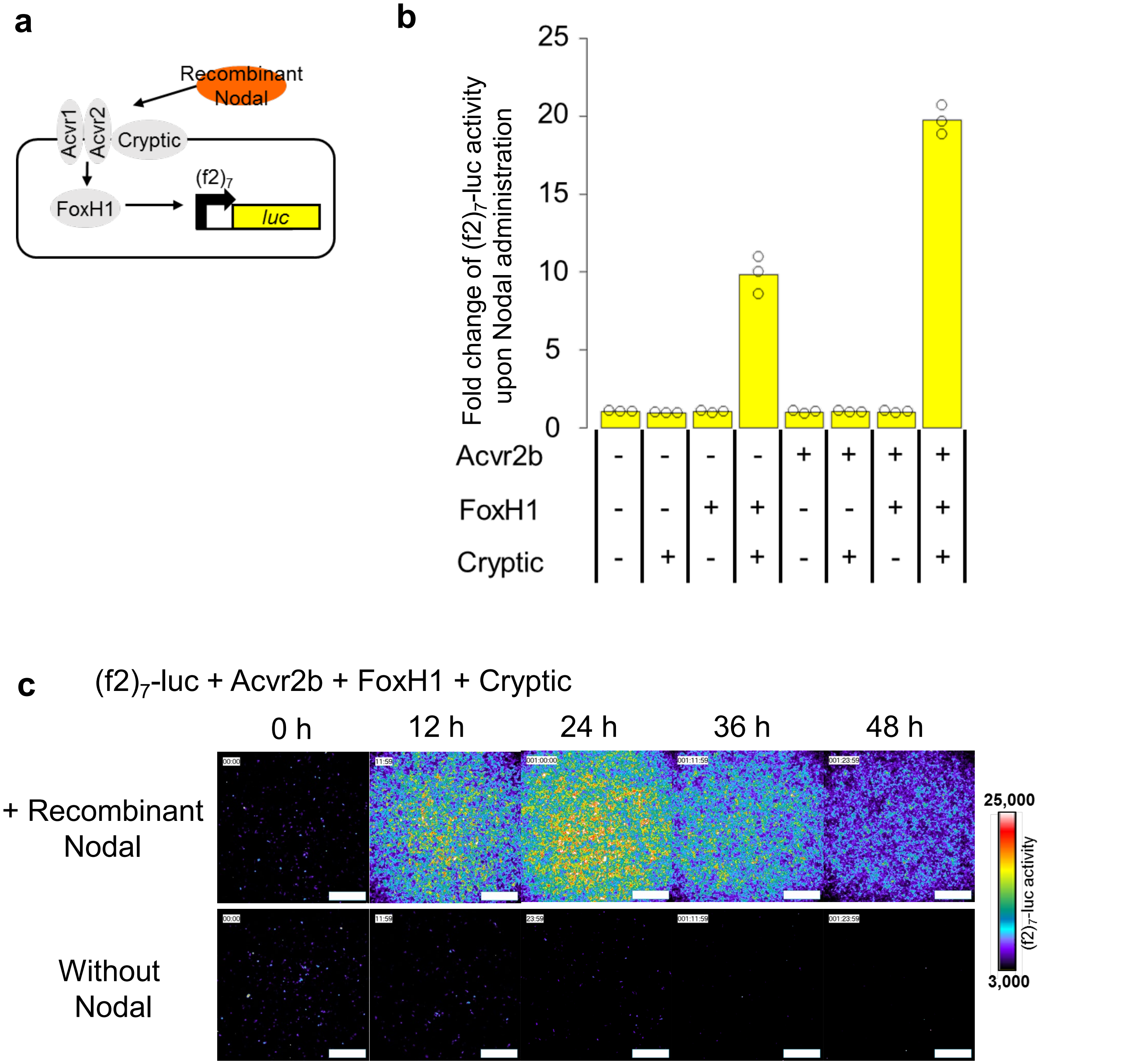

Supplementary figure 1. *Cryptic*, *FoxH1* and *Acvr2b* are required for HEK293 cells to efficiently respond to Nodal stimulation.
(**a**) Different combinations of *Cryptic* (co-receptor), *FoxH1* (transcription factor) and *Acvr2b* (receptor) were introduced into HEK293 cells, and the Nodal signaling activities were monitored with the $(f2)_7$-luc reporter. (**b**) The $(f2)_7$-luc activities in the absence and presence of 10 nM recombinant Nodal were measured, and the fold-changes are shown. Data are means and individual points (n=3). (**c**) Time-lapse imaging of the HEK293 cells engineered with *(f2)7-luc*, *CAG-Acvr2b*, *CAG-FoxH1* and *CAG-Cryptic* upon 10 nM Nodal stimulation. Scale bars: 400 µm. Source data are provided as a Source Data file (b).

Supplementary Figure 2

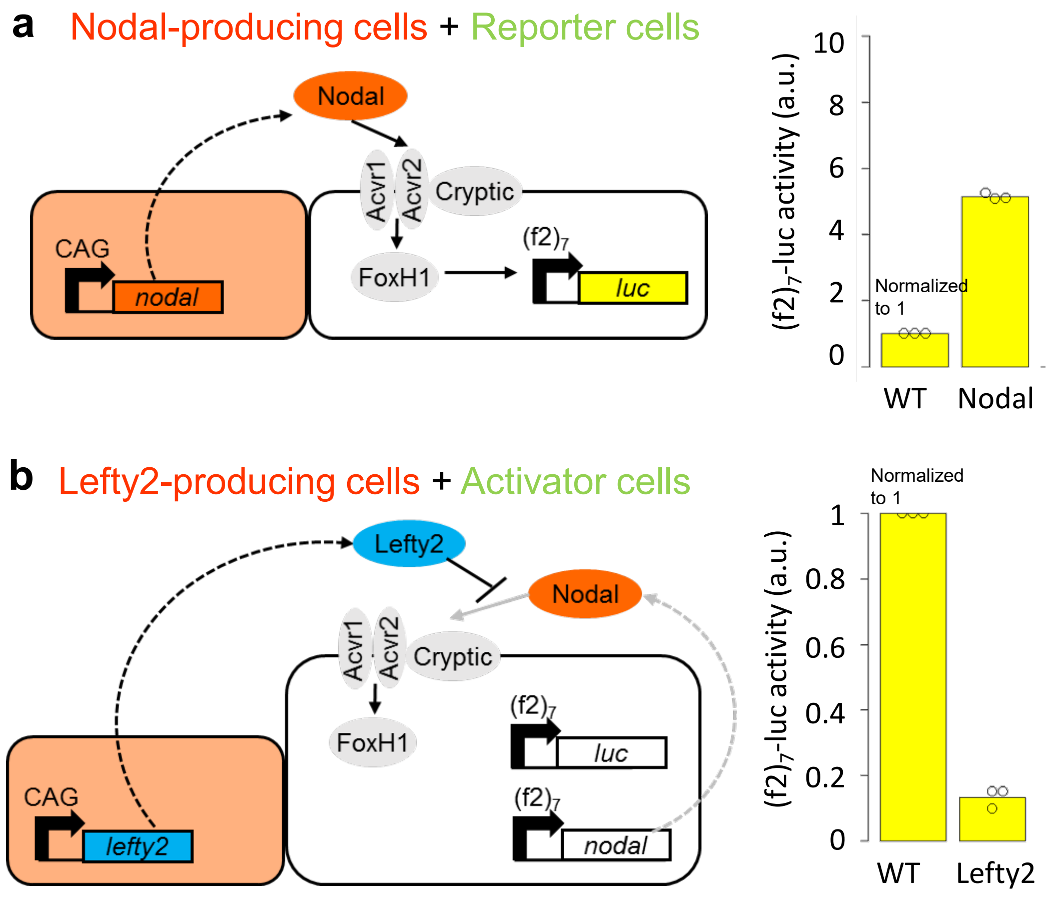

Supplementary figure 2. Co-culture with Nodal- or Lefty2-producing cells.
(**a**) The Nodal-producing cells or wild-type cells were co-cultured with the reporter cells, and the $(f2)_7$-luc activity of the reporter cells was measured 48 hours later. (**b**) The Lefty2-producing cells or wild-type cells were co-cultured with the activator cells, and the $(f2)_7$-luc activity of the activator cells was measured 48 hours later. Data are means and individual points (n=3). Source data are provided as a Source Data file.

Supplementary Figure 3

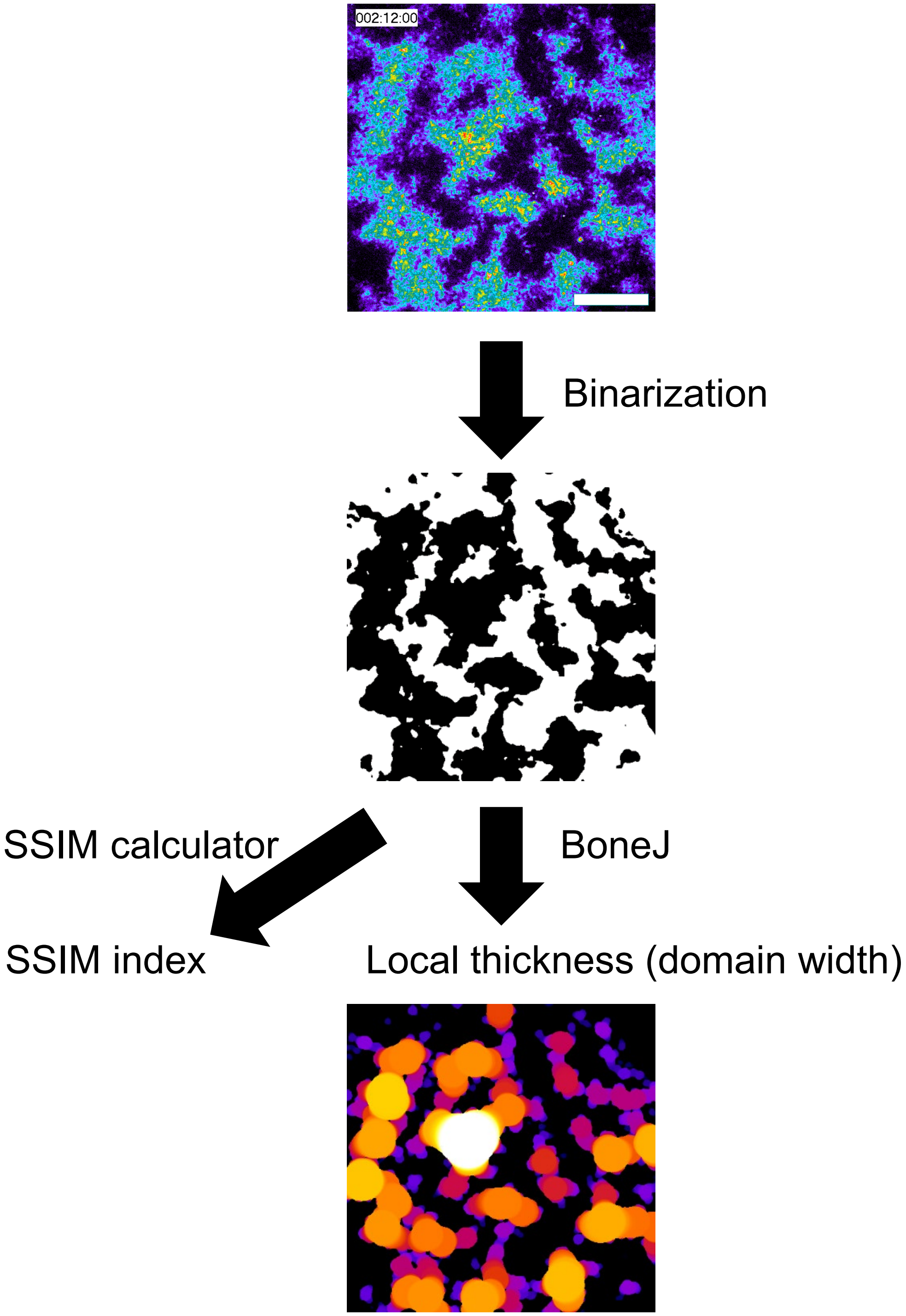

Supplementary figure 3. A scheme of pattern analyses.
Raw luciferase images were binarized (threshold = the median of luminescence distribution). Then the structure similarity (SSIM) index and local thickness were calculated with the imageJ plugins, SSIM_INDEX and BoneJ, respectively. The average positive/negative domain width was defined as the average value of the local thickness.

Supplementary Figure 4

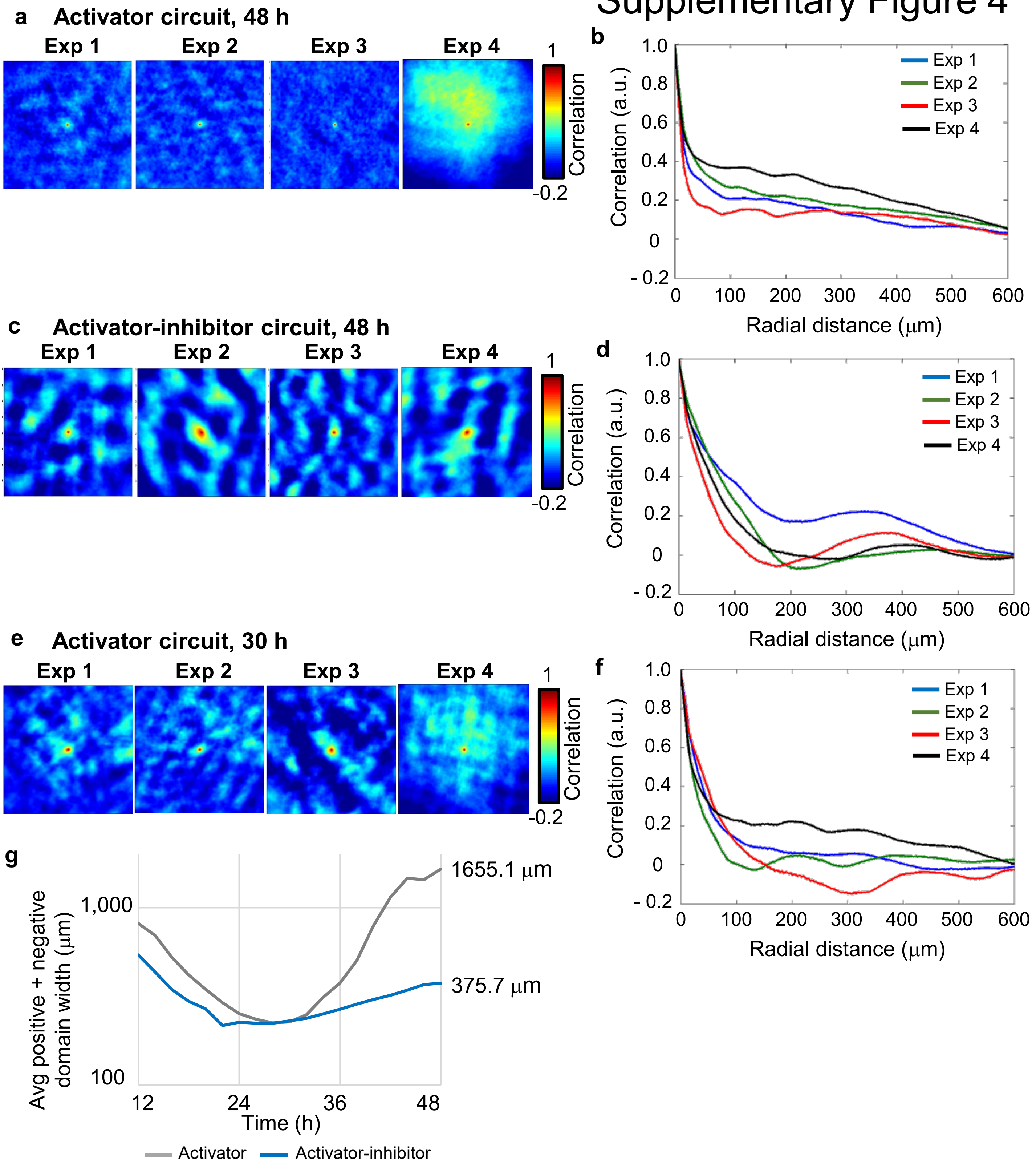

Supplementary figure 4. Spatial correlation analysis of the synthetic pattern.
(**a**) The spatial correlation was calculated for the image of the activator cell line at 48 hours. (**b**) The correlation function shown in (a) was radially averaged and plotted. (**c**) The spatial correlation was calculated for the pattern of the activator-inhibitor cell line at 48 hours. (**d**) The correlation function shown in (c) was radially averaged and plotted, revealing a second peak at around 400 μm. (**e**) The spatial correlation was calculated for the image of the activator cell line at 30 hours. (**f**) The correlation function shown in (e) was radially averaged and plotted. To perform a statistical test, the integral of radial correlation between 150 - 250 μm was subtracted from the integral of radial correlation between 300 - 400 μm. The calculated value for Activator 48 h (b), Activator-inhibitor 48 h (d) and Activator 30 h (f) was -1.88 ± 0.55, 1.75 ± 0.74 and, -1.16 ± 0.37 respectively (n = 4, mean ± s.e.m). The two-sided Wilcoxon rank sum test was performed to compare Activator-inhibitor 48 h and Activator 30 h ($p = 0.029$). (**g**) The sum of the average positive domain width and negative domain width at each time point of Experiment 1, 2 and 3. Source data are provided as a Source Data file (g).

Supplementary Figure 5

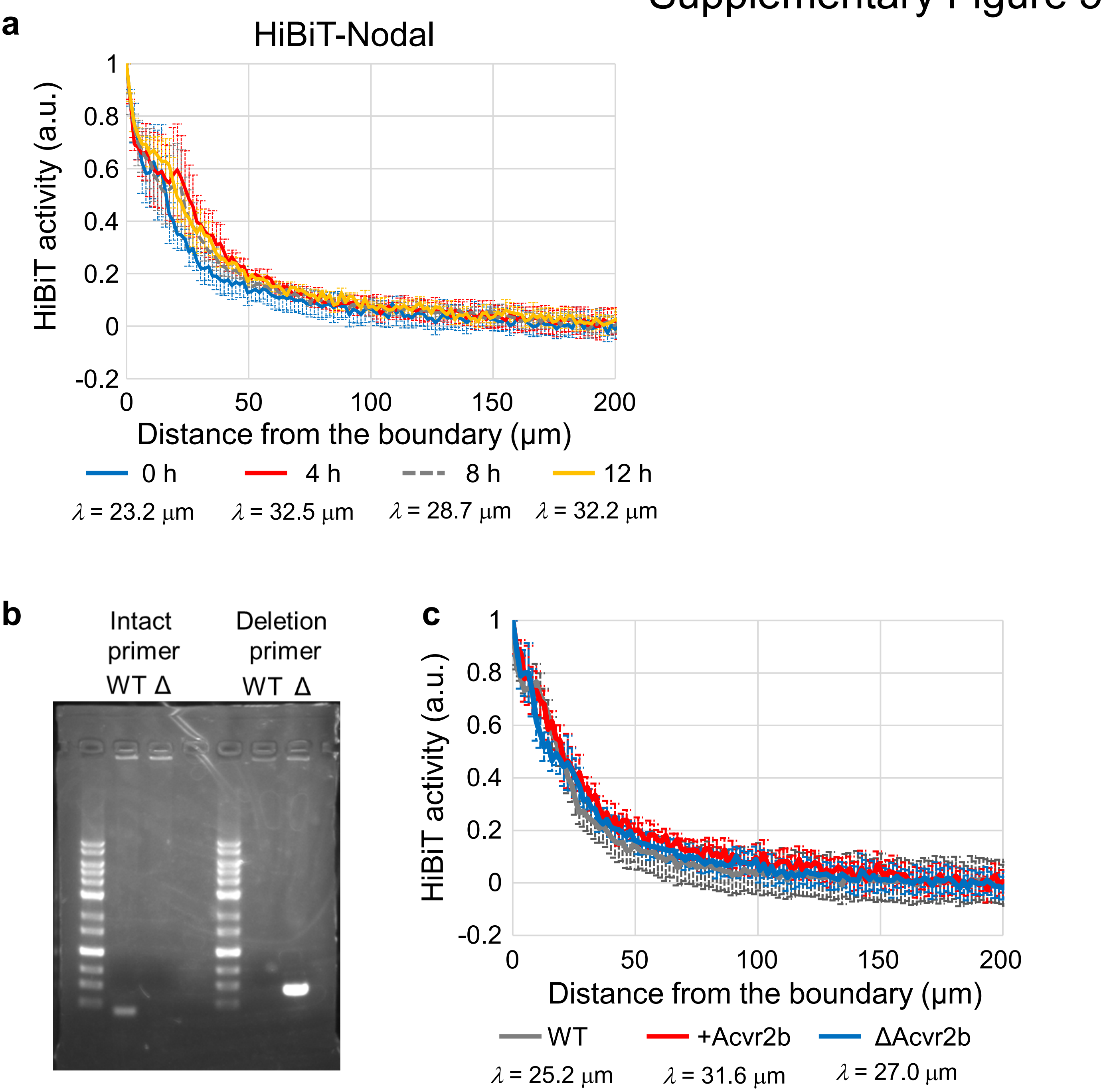

Supplementary figure 5. Extracellular distribution of Nodal.
(**a**) Quantified distribution profiles of HiBiT-Nodal at 0, 4, 8 and 12 hours after the addition of the LgBiT and substrate in the culture insert assay. The data at 8 hours is also shown in Fig. 2e. (**b**) The human *Acvr2b* gene (86 - 1459 bp in ORF) was deleted from the HEK293 genome with CRISPR/Cas9. The deletion of *Acvr2b* was confirmed by PCR. WT: Genome template from wild-type HEK293 cells. Δ: Genome template form the *Acvr2b* deletion mutant. Intact primer: a primer pair that detects wild-type *Acvr2b*. Deletion primer: a primer pair that detects an *Acvr2b* deletion mutant. See also Supplementary table 1. (**c**) The effect of Acvr2b on the distribution range of HiBiT-Nodal. HiBiT-Nodal was introduced into the wild-type cells, *Acvr2b*-overexpressing cells and *Acvr2b*-deleted cells. These ligand cells were co-cultured with the corresponding receptor cells (wild-type cells, *Acvr2b*-overexpressing cells and *Acvr2b*-deleted cells, respectively) in the culture insert assay. Source data are provided as a Source Data file (a, c).

Supplementary Figure 6

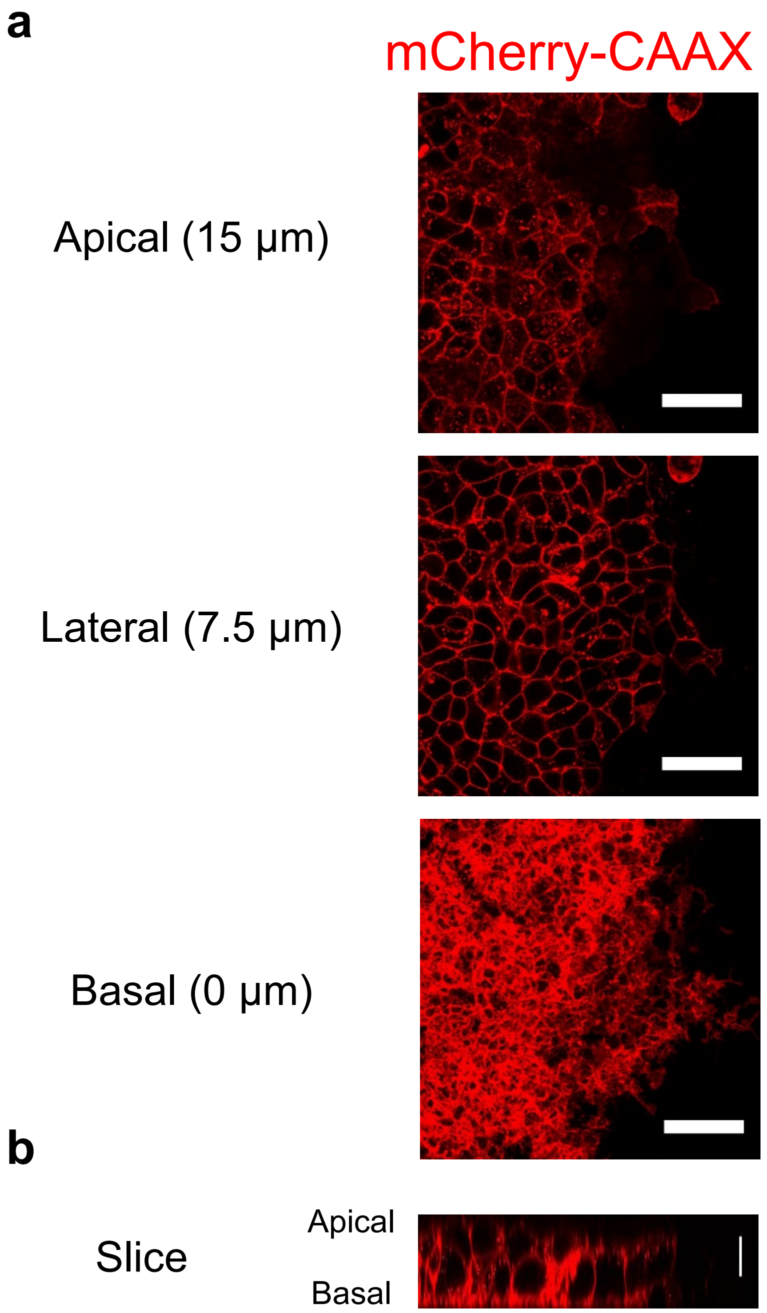

Supplementary figure 6. A z-stack view of ligand-producing cells.
(**a,b**) The ligand cells (labeled with mCherry-CAAX) in the culture insert assay were imaged with a confocal microscope. The basal side was defined as the point where the cell membranes showed a dense structure spreading on the dish. The lateral side and apical side were defined as the points 7.5 μm and 15 μm higher than the basal side, respectively. Scale bars: 50 μm (a); 10 μm (b).

Supplementary Figure 7

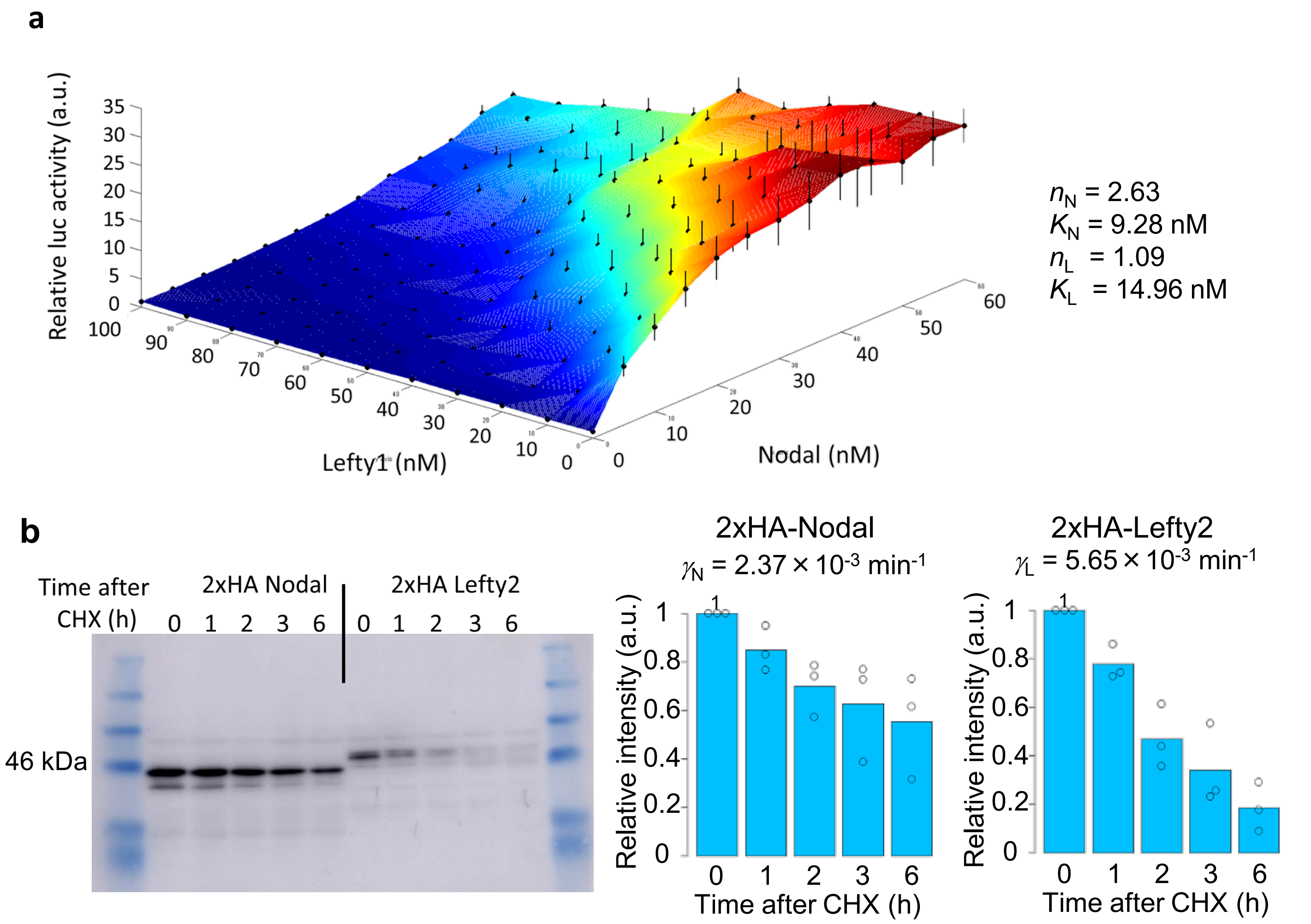

Supplementary figure 7. Parameter measurements.
(**a**) Measurements of the signal response curve. The reporter cells containing *(f2)*$_7$*-luc*, *Cryptic* and *FoxH1* were treated with different concentrations of recombinant Nodal and Lefty1. The (f2)$_7$-luc activities were measured 2 days later. Data are means and s.e.m. (n=3). (**b**) Measurements of the degradation rates. The protein amounts of the HA-tagged Nodal (43.7 kDa) and HA-tagged Lefty2 (44.6 kDa) were measured at each time point after the addition of cycloheximide (CHX, 50 µg/ml). Left: A representative gel image of the immunoblotting. Center and right: The quantification of (b). Data are means and individual points (n=3). Note that the extracellular and intracellular proteins were not distinguished and that extracellular proteins floating in the medium were ignored in this measurement. Using the degradation rates γ and the characteristic distances λ measured in Fig. 2e, the diffusion rates D of Nodal and Lefty were estimated as $D_N = 1.96$ µm^2 min^{-1} and $D_L = 56.39$ µm^2 min^{-1}. Source data are provided as a Source Data file (b).

Supplementary Figure 8

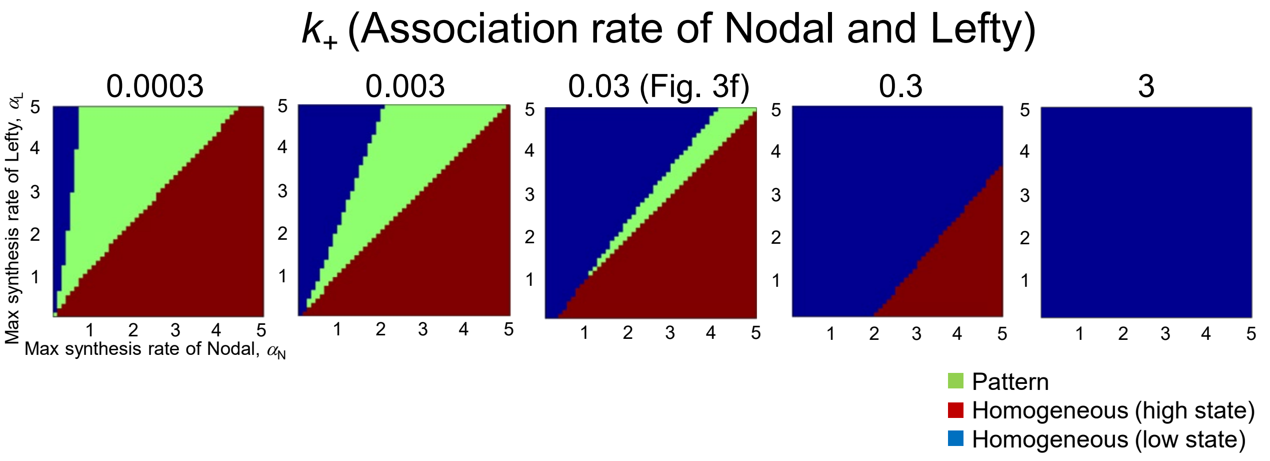

Supplementary figure 8. The effect of k_+ on pattern formation.

The competitive inhibition + direct inhibition model was simulated in one dimension with different values of k_+, the association rate of Nodal and Lefty. The parameter region for pattern formation (green) is shown for each k_+. The phase diagram at $k_+ = 0.03$ is also shown in Fig. 3f.

Supplementary Figure 9

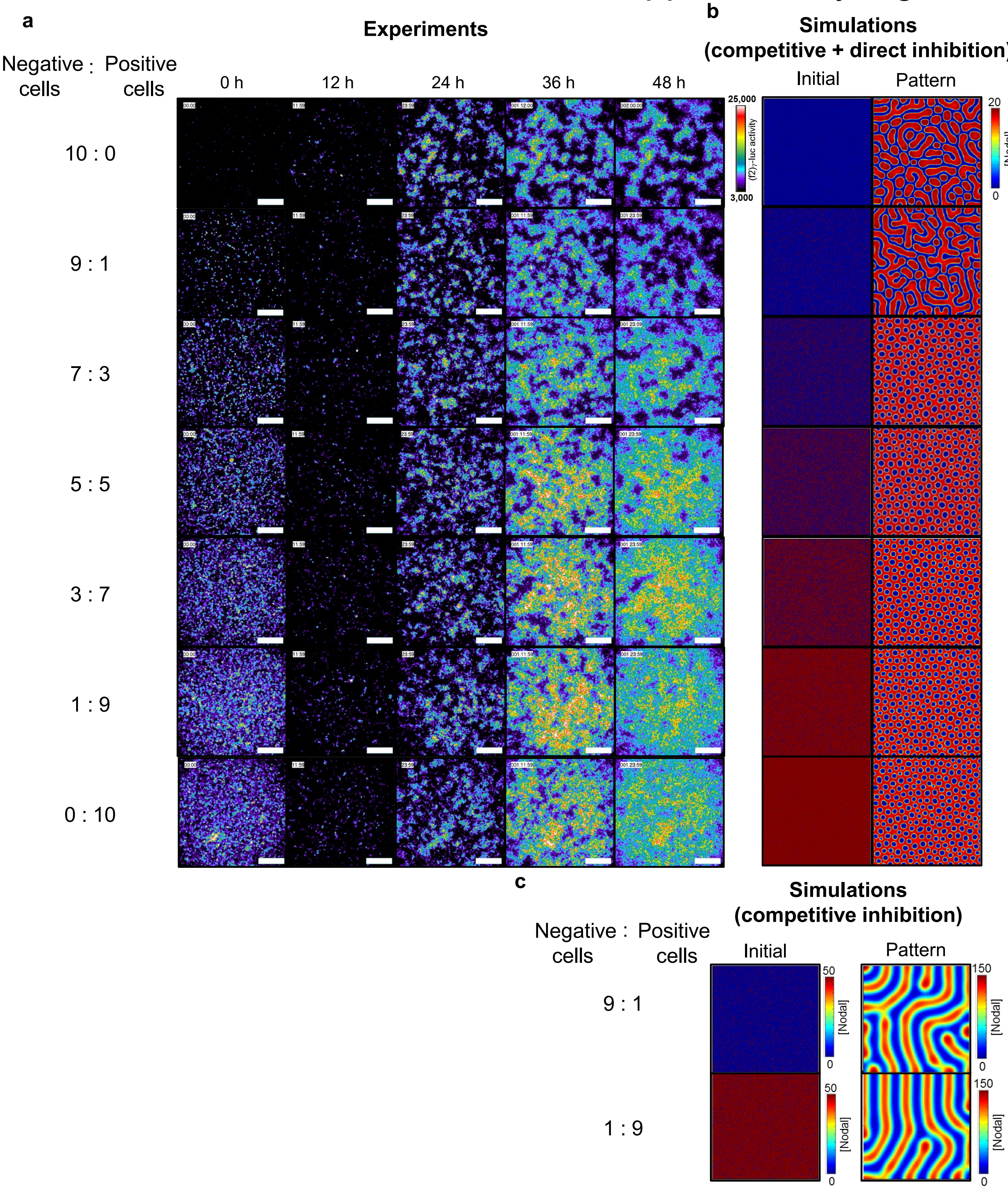

Supplementary figure 9. Varying the initial condition**.**
(**a**) The reporter-positive cells and negative cells were prepared by pretreatment with 20 nM recombinant Nodal and 10 μM SB431542 (Tgf-β inhibitor), respectively. The positive and negative cells were mixed at indicated ratios to vary the initial condition. Scale bars: 400 μm. (**b**) Simulations of the competitive inhibition + direction inhibition model with corresponding initial conditions. The ratios of 0:10 and 10:0 mean 1:99 and 99:1, respectively. The initial image and the resulting pattern are shown. (**c**) Simulations of the competitive inhibition model with indicated initial conditions.

Supplementary Figure 10

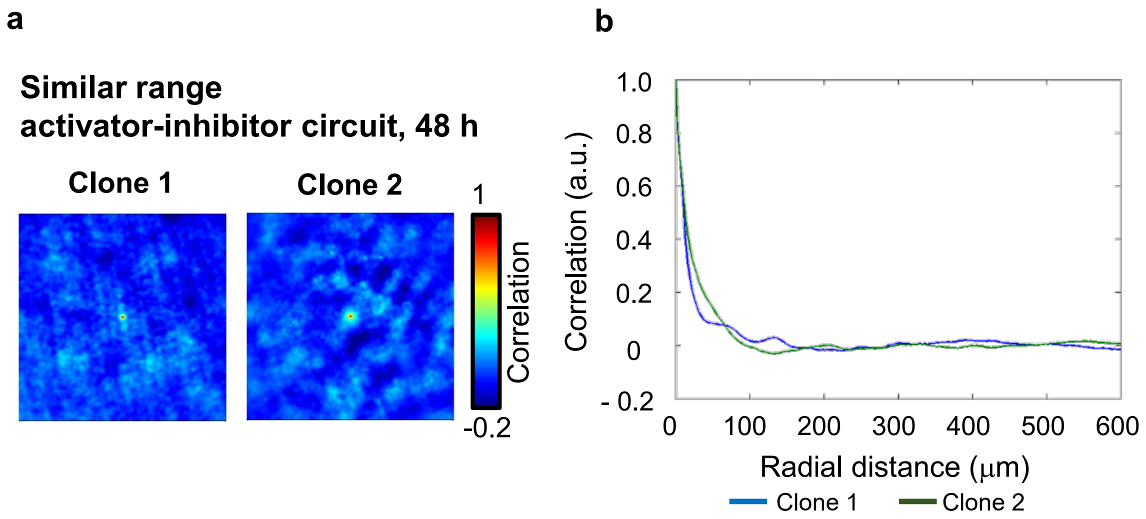

Supplementary figure 10. Spatial correlation analysis of the similar range activator-inhibitor circuit. (**a**) The spatial correlation was calculated for the image of the cells engineered with the similar range activator-inhibitor circuit. (**b**) The correlation function shown in (a) was radially averaged and plotted.
