# Supplementary material for: Synthetic mammalian pattern formation driven by differential diffusivity of Nodal and Lefty
Source: Nat Commun. 2018 Dec 21;9:5456. doi: 10.1038/s41467-018-07847-x (PMC6303393; doi:10.1038/s41467-018-07847-x)
Supplement: Supplementary file 3 — Description of Additional Supplementary Files [file 41467_2018_7847_MOESM3_ESM.docx]

**Title:** Supplementary movie 1.
**Description:** The time-lapse imaging of the HEK293 cells engineered with the activator circuit and that with the activator-inhibitor circuit. Scale bar: 400 μm.
