# Supplementary material for: Synthetic mammalian pattern formation driven by differential diffusivity of Nodal and Lefty
Source: Nat Commun. 2018 Dec 21;9:5456. doi: 10.1038/s41467-018-07847-x (PMC6303393; doi:10.1038/s41467-018-07847-x)
Supplement: Supplementary file 6 — Reporting Summary [file 41467_2018_7847_MOESM6_ESM.pdf]

nature research

Corresponding author(s): Miki Ebisuya

Reporting Summary

Nature Research wishes to improve the reproducibility of the work that we publish. This form provides structure for consistency and transparency in reporting. For further information on Nature Research policies, see Authors & Referees and the Editorial Policy Checklist.

Statistical parameters

When statistical analyses are reported, confirm that the following items are present in the relevant location (e.g. figure legend, table legend, main text, or Methods section).

n/a	Confirmed	
☐	☒	The exact sample size (*n*) for each experimental group/condition, given as a discrete number and unit of measurement
☐	☒	An indication of whether measurements were taken from distinct samples or whether the same sample was measured repeatedly
☐	☒	The statistical test(s) used AND whether they are one- or two-sided<br>*Only common tests should be described solely by name; describe more complex techniques in the Methods section.*
☒	☐	A description of all covariates tested
☒	☐	A description of any assumptions or corrections, such as tests of normality and adjustment for multiple comparisons
☐	☒	A full description of the statistics including central tendency (e.g. means) or other basic estimates (e.g. regression coefficient) AND variation (e.g. standard deviation) or associated estimates of uncertainty (e.g. confidence intervals)
☐	☒	For null hypothesis testing, the test statistic (e.g. *F*, *t*, *r*) with confidence intervals, effect sizes, degrees of freedom and *P* value noted<br>*Give P values as exact values whenever suitable.*
☒	☐	For Bayesian analysis, information on the choice of priors and Markov chain Monte Carlo settings
☒	☐	For hierarchical and complex designs, identification of the appropriate level for tests and full reporting of outcomes
☒	☐	Estimates of effect sizes (e.g. Cohen's *d*, Pearson's *r*), indicating how they were calculated
☐	☒	Clearly defined error bars<br>*State explicitly what error bars represent (e.g. SD, SE, CI)*

Our web collection on statistics for biologists may be useful.

Software and code

Policy information about availability of computer code

Data collection	*Provide a description of all commercial, open source and custom code used to collect the data in this study, specifying the version used OR state that no software was used.*
Data analysis	R studio for bee swarm plots (e.g. Fig. 4f), Microsoft Excel 2013 for the other data analyses

For manuscripts utilizing custom algorithms or software that are central to the research but not yet described in published literature, software must be made available to editors/reviewers upon request. We strongly encourage code deposition in a community repository (e.g. GitHub). See the Nature Research guidelines for submitting code & software for further information.

Data

Policy information about availability of data

All manuscripts must include a data availability statement. This statement should provide the following information, where applicable:

- Accession codes, unique identifiers, or web links for publicly available datasets
- A list of figures that have associated raw data
- A description of any restrictions on data availability

The data in this paper is available from the corresponding author upon reasonable request.

Field-specific reporting

Please select the best fit for your research. If you are not sure, read the appropriate sections before making your selection.

☒ Life sciences ☐ Behavioural & social sciences ☐ Ecological, evolutionary & environmental sciences

For a reference copy of the document with all sections, see nature.com/authors/policies/ReportingSummary-flat.pdf

Life sciences study design

All studies must disclose on these points even when the disclosure is negative.

Sample size	At least three independent experiments were performed for each data. The number of replicates was determined according to the standard experiments reported in literature.
Data exclusions	No data was excluded.
Replication	All experiments were repeated at least three times.
Randomization	Randomization was not performed.
Blinding	Blinding was not performed.

Reporting for specific materials, systems and methods

Materials & experimental systems

n/a	Involved in the study
☒	☐ Unique biological materials
☐	☒ Antibodies
☐	☒ Eukaryotic cell lines
☒	☐ Palaeontology
☒	☐ Animals and other organisms
☒	☐ Human research participants

Methods

n/a	Involved in the study
☒	☐ ChIP-seq
☐	☒ Flow cytometry
☒	☐ MRI-based neuroimaging

Antibodies

Antibodies used	mouse anti-HA antibody (cat.no. 901501, Biolegend), 1/3,000<br>sheep anti-mouse antibody (NA931, GE Healthcare), 1/8,000
Validation	mouse anti-HA antibody (https://www.biolegend.com/en-us/products/purified-anti-ha-11-epitope-tag-antibody-11374), anti-mouse antibody (https://www.biocompare.com/9776-Antibodies/323128-Amersham-ECL-Mouse-IgG-HRP-linked-whole-Ab-from-sheep/)

Eukaryotic cell lines

Policy information about cell lines

Cell line source(s)	293AD from cell biolabs, inc.
Authentication	No authentication of the cell lines was performed.
Mycoplasma contamination	The 293AD cells we purchased were tested free of microbial contamination.
Commonly misidentified lines (See ICLAC register)	None

Flow Cytometry

Plots

Confirm that:

☒ The axis labels state the marker and fluorochrome used (e.g. CD4-FITC).

☒ The axis scales are clearly visible. Include numbers along axes only for bottom left plot of group (a 'group' is an analysis of identical markers).

☒ All plots are contour plots with outliers or pseudocolor plots.

☒ A numerical value for number of cells or percentage (with statistics) is provided.

Methodology

Sample preparation	Cells cultured on 60 mm dish were washed once with 1xPBS, and processed with Trypsin/EDTA for 2 min at 37 degrees. Then the medium was added, and the cells were centrifuged for 2 min at x300 g. The supernatant was aspirated, and 2 ml medium was added to the pellet. Then, the cells in medium were used for the measurement.
Instrument	JSAN cell sorter (Bay bioscience)
Software	Collection: AppSan, Analysis: Flowjo
Cell population abundance	10,000 cells were measured for each analysis. Sorting was not performed.
Gating strategy	Approximately 80% of the collected cells were gated with FSC/SSC intensities and the cell debris were removed. The same gate was used for the control and sample.

☐ Tick this box to confirm that a figure exemplifying the gating strategy is provided in the Supplementary Information.
